# Increased Cortical Porosity and Reduced Trabecular Density Are Not Necessarily Synonymous With Bone Loss and Microstructural Deterioration

**DOI:** 10.1002/jbm4.10078

**Published:** 2018-10-04

**Authors:** Roger Zebaze, Elizabeth J Atkinson, Yu Peng, Minh Bui, Ali Ghasem‐Zadeh, Sundeep Khosla, Ego Seeman

**Affiliations:** ^1^ Departments of Medicine and Endocrinology Austin Health University of Melbourne Melbourne Australia; ^2^ Straxcorp Pty Ltd Melbourne Australia; ^3^ Mayo Clinic Rochester MN USA; ^4^ Centre for Epidemiology and Biostatistics School of Population and Global Health University of Melbourne Melbourne Australia; ^5^ Mary Mackillop Institute for Health Research Australian Catholic University Melbourne Australia

**Keywords:** BONE MINERAL DENSITY, CORTICAL POROSITY, MICROSTRUCTURAL DETERIORATION, TRABECULAR DENSITY

## Abstract

Absolute values of cortical porosity and trabecular density are used to estimate fracture risk, but these values are the net result of their growth‐related assembly and age‐related deterioration. Because bone loss affects both cortical and trabecular bone, we hypothesized that a surrogate measure of bone fragility should capture the age‐related deterioration of both traits, and should do so independently of their peak values. Accordingly, we developed a structural fragility score (SFS), which quantifies the increment in distal radial cortical porosity and decrement in trabecular density relative to their premenopausal mean values in 99 postmenopausal women with forearm fractures and 105 controls using HR‐pQCT. We expressed the results as odds ratios (ORs; 95% CI). Cortical porosity was associated with fractures in the presence of deteriorated trabecular density (OR 2.30; 95% CI, 1.30 to 4.05; *p* = 0.004), but not if trabecular deterioration was absent (OR 0.96; 95% CI, 0.50 to 1.86; *p* = 0.91). Likewise, trabecular density was associated with fractures in the presence of high cortical porosity (OR 3.35; 95% CI, 1.85 to 6.07; *p* < 0.0001), but not in its absence (OR 1.60; 95% CI, 0.78 to 3.28; *p* = 0.20). The SFS, which captures coexisting cortical and trabecular deterioration, was associated with fractures (OR 4.52; 95% CI, 2.17 to 9.45; *p* < 0.0001). BMD was associated with fracture before accounting for the SFS (OR 5.79; 95% CI, 1.24 to 27.1; *p* = 0.026), not after (OR 4.38; 95% CI, 0.48 to 39.9; *p* = 0.19). The SFS was associated with fracture before (OR 4.67; 95% CI, 2.21 to 9.88) and after (OR 3.94; 95% CI, 1.80 to 8.6) accounting for BMD (both *p*s < 0.0001). The disease of bone fragility is captured by cortical and trabecular deterioration: A measurement of coexisting cortical and trabecular deterioration is likely to identify women at risk for fracture more robustly than absolute values of cortical porosity, trabecular density, or BMD. © 2018 The Authors. *JBMR Plus* Published by Wiley Periodicals, Inc. on behalf of the American Society for Bone and Mineral Research

## Introduction

Fracture risk increases as BMD decreases.^1^ However, the diagnostic threshold for osteoporosis, a BMD *T*‐score at the femoral neck of −2.5 SDs, is insensitive; it fails to detect most postmenopausal women with fractures because they have osteopenia (*T*‐score, −2.5 to −1.0 SD) or so‐called normal BMD (> −1.0 SD).^2^, ^3^

A plausible reason for a high fracture risk in postmenopausal women independent of BMD is the presence of microstructural deterioration.^4^, ^5^, ^6^ All women lose bone as their age advances. In women with a high peak BMD, bone loss may only decrease BMD into the low normal or osteopenic range, while bone fragility increases disproportionately to the bone loss producing it and the modest reduction in BMD. The reason for this, as shown in ex vivo studies of bone specimens, is an increase in void volume (porosity) of a compact structure like cortical bone compromises strength as a seventh‐power function of porosity, whereas an increase in void volume of an already porous structure like trabecular bone compromises bone strength as a third‐power function of trabecular density.^7^

Quantifying microstructure is now feasible,^8^ and measurement of cortical porosity and trabecular density does distinguish women with osteopenia with prevalent fractures from women with osteopenia without fractures.^4^, ^5^, ^6^ However, these findings have not been replicated in all studies,^9^, ^10^, ^11^, ^12^ perhaps because of unresolved challenges in image acquisition and analyses.

For example, regarding image acquisition, unbalanced remodeling upon intracortical canal surfaces increases cortical porosity. The porosity and the trabecularized fragments of cortex adjacent to the medullary canal are erroneously allocated to the medullary canal void volume by the image segmentation algorithm (rather than being confined to a cortico–trabecular transitional zone). The cortical porosity is measured as part of a seemingly expanded medullary canal, leading to an underestimate of the age‐related increase in cortical porosity. The cortical fragments residing within the seemingly expanded medullary canal look like trabeculae, and are measured as part of the trabecular compartment, thus overestimating trabecular density and leaving women at high risk for fracture undetected.^13^, ^14^

The second concern is the use of term “porosity.” Porosity is not always synonymous with microstructural deterioration, nor is it always the consequence of bone loss or a cause of bone fragility. Absolute values of cortical porosity are the net result of the growth‐related assembly of Haversian canals that are seen as “pores” in cortical cross‐sections, and any age‐related focal enlargement of the canals produced by unbalanced remodeling.^15^, ^16^, ^17^, ^18^, ^19^ The canals are part of the osteonal microstructure that confers strength to bone by obstructing microcrack propagation.^20^, ^21^ If this “porosity” is erroneously attributed to bone loss and included in the calculation of porosity, women may be misclassified as being at risk for fracture.

The third concern is the need to measure both cortical and trabecular compartments. Finding deficits in both increases the likelihood that the deficits are based on bone loss because bone loss is global: It affects both cortical and trabecular compartments.^13^, ^22^ Measuring only one compartment may be misleading because this is likely because of errors in positioning of the region of interest (ROI) as reported elsewhere.^23^ In brief, in an individual, a similar volume of mineralized bone matrix assembles adjacent cross‐sections along a bone, what differs from cross‐section to cross‐section is the volume of void (medullary canal and cortical porosity) used to assemble larger and smaller cross‐sections.^23^, ^24^

**Figure 1:**
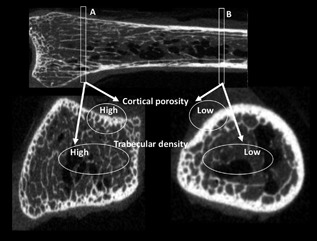
If the region of interest (ROI) is too distal (position A), cortical porosity will be high, suggesting bone loss, whereas high trabecular density suggests no bone loss. If the ROI is too far proximally along the shaft, the porosity will be low, suggesting no bone loss, but low trabecular density suggests bone loss. Positioning error may produce misleading results (see text).

As illustrated in Fig. 1, most of the mineralized bone matrix distally is assembled as trabecular bone; the cortices are thin and porous. Positioning the ROI too far distally results in a trait dissociation with high cortical porosity suggesting bone loss, but high trabecular density suggesting otherwise. Proximally, most of the same amount of mineralized bone matrix is assembled as cortical bone of low porosity, trabeculae are few or absent. Positioning the ROI too far proximally also results in a trait dissociation with low trabecular density suggesting bone loss, but low cortical porosity suggesting otherwise.

These concerns led us to develop a surrogate of bone fragility, a structural fragility score (SFS), a single measurement on a continuous scale that measures the concurrent deterioration in cortical porosity and trabecular density relative to their respective means in premenopausal women. The measurement is relatively free of growth‐related determinants of bone strength and errors in positioning of the ROI. We hypothesized that the SFS would identify women with prevalent fractures, would do so with greater sensitivity and specificity than the absolute values of cortical porosity or trabecular density, and would do so independently of BMD; the predictive strength of BMD would be dependent on microstructural deterioration captured by the SFS.

## Subjects and Methods

### Participants

As reported, we matched 99 postmenopausal women aged over 50 years with a distal forearm fracture with 105 controls from an age‐stratified random sample from Olmsted County, Minnesota, USA.^5^ Fracture occurred 7 (3 to 13) months (median interquartile range [IQR]) before measurement of microstructure. Fragility fracture was defined on the basis of moderate trauma from a fall from standing height or less. Controls had no history of a fracture after 35 years of age. The cohort was >96% white. The study was approved by the Mayo Clinic Institutional Review Board (Rochester, MN, USA), and the present analysis was based on deidentified data.

### Imaging

Images at the ultradistal radius (on the unfractured side) were obtained using HR‐pQCT (XtremeCT; Scanco Medical AG, Brüttisellen, Switzerland) with the X‐ray source potential set to 60 kVp and a current of 900 μA. Stacks of 110 images (9.02 mm) were acquired with a voxel size of 82 µm.^8^ Quality control was monitored by daily scans of phantoms (hydroxyapatite [HA] rods; QRM, Moehrendorf, Germany). Cortical porosity and trabecular density were measured using StrAx1.0 (StraxCorp, Melbourne, Australia).^13^, ^14^ StrAx1.0 is limited to the proximal 49 slices, where cortices at the thickest allow a more robust quantification of porosity. Femoral neck (FN) BMD was measured by DXA (Lunar Prodigy; GE Healthcare, Piscataway, NJ, USA).

### Derivation of the SFS

**Figure 2:**
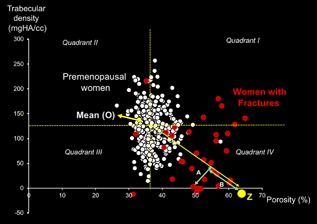
Trabecular density as a function of cortical porosity in healthy premenopausal women (white dots) and postmenopausal women with fragility fractures (red dots). The mean of each trait in premenopausal women is denoted by “O,” the intersection of the horizontal and vertical hatched lines. The line from O to Z is the mean of all the slopes of lines from 0 to each red dot. Quadrants I to IV depict different combinations of high and low trabecular density and cortical porosity (see text).

We plotted trabecular density as a function of cortical porosity in 324 healthy ambulant premenopausal women aged 20 to 40 years and 33 postmenopausal women aged 50 to 90 years with fragility fractures in Melbourne, Australia (Fig. 2). Individuals with cortical porosity and trabecular in quadrant I (high porosity, high trabecular density), quadrant II (low porosity, high trabecular density), or quadrant III (low porosity, low trabecular density) are unlikely to reflect bone loss because bone loss is global and affects both compartments, increasing cortical porosity and decreasing trabecular density. Thus, finding values in quadrant IV (high porosity, low trabecular density) is consistent with bone loss in both compartments, causing bone fragility. Most women with fractures have microstructure in this quadrant.

The SFS is derived by quantifying the coexisting absolute increment in cortical porosity and the absolute decrement trabecular density, relative to their peak values in premenopausal women as a continuous variable. The *x* and *y* coordinates at O are the mean of each trait in premenopausal women. The regression line U from O in the direction of increasing porosity and decreasing trabecular density is the average of all the slopes between point O and all *x* and *y* coordinates, representing absolute values in women with fractures. The length of U captures the absolute deterioration of an individual's cortical and trabecular bone while the slope captures deterioration in porosity relative to deterioration in trabecular density. The point Z along U identifies the maximum deterioration.

For individuals with *x* and *y* coordinates on U, length B measures the deterioration in both traits. The closer an individual's *x* and *y* coordinates are to U (distance A), the more likely there will be coexisting cortical and trabecular bone loss. Values above U reflect greater cortical than trabecular deficits relative to the young normal mean. Values below U reflect the reverse. For these individuals, the perpendicular length A plus length B to Z is a measure of the deterioration of that individual's bone microstructure. The lower (A + B), the closer to Z, the more severe the microstructural deterioration. For ease of comprehension, we express SFS as [100−(A + B)]; so the greater the SFS, the greater the cortical and trabecular deterioration and the higher the fracture risk. The precision of the SFS tests acquisition, repositioning, and coregistration in 15 women having three measurements at the distal radius and was 1.12% expressed as the root mean square of the coefficient of variation.

### Statistical analysis

Thresholds used to calculate the odds ratios (ORs) for fracture were 90^th^ centile for cortical porosity, 5^th^ centile for trabecular density, −2.5 SD for BMD, and 70 for the SFS corresponding to the 90^th^ percentile in premenopausal women. ORs were obtained from logistic regression models before and after adjustment for covariates. A *p* < 0.05 (two‐tailed) denoted statistical significance.

## Results

**Figure 3:**
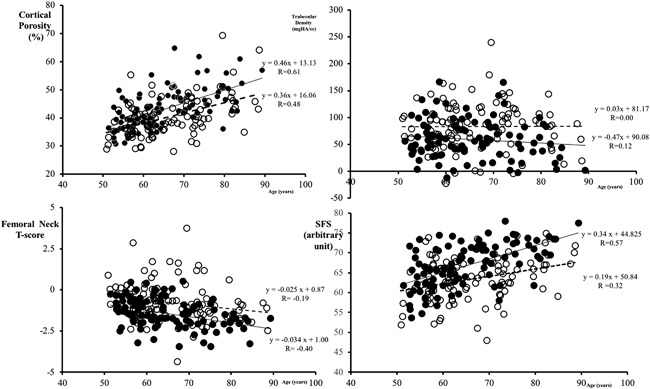
Advancing age in women with fractures and controls was associated with increments in cortical porosity (*r* = 0.61 and 0.48, respectively; both *p*s < 0.0001), not trabecular density, decrements in BMD (*r* = −0.4, *p* < 0.0001, −0.19, respectively, *p* = 0.05), and increments in structural fragility score (SFS; *r* = 0.57 and 0.32, respectively; both *p*s < 0.0001). The increment in SFS was greater in women with fractures than in controls (*p* = 0.02).

Among the women with forearm fractures, 10 (10.1%) had osteoporosis, 59 (59.6%) had osteopenia, and 30 (30.3%) had normal BMD. Women with prevalent fractures did not differ in age, but had higher cortical porosity, lower trabecular density, lower BMD, and higher SFS than controls (Table 1). There were significant age‐related increments or decrements in cortical porosity, BMD, and the SFS, but not in trabecular density (Fig. 3). The age‐related increment in the SFS was greater in women with fractures than in controls (*p* = 0.02).

**Table 1:** Characteristics of All Women With and Without Prevalent Fractures

	Fracture		Nonfracture		
	*N* = 99		*N* = 105		
	Median	IQR	Median	IQR	*p* value
Age (years)	63.00	14.00	62.00	14.00	0.392
Cortical porosity (%)	42.20	9.20	39.50	7.80	0.003
Trabecular density (mg HA/cc)	55.60	45.50	85.90	46.80	<0.0001
Femoral neck BMD *T*‐score	−1.50	1.20	−0.95	1.46	0.0001
SFS (arbitrary unit)	61.60	28.10	51.70	20.40	0.0001

IQR = interquartile range; SFS = structural fragility score.

### Associations between microstructure and fractures depend on coexisting deficits in cortical and trabecular bone

**Figure 4:**
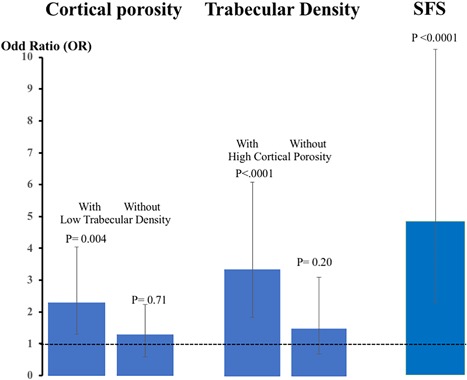
Cortical porosity was associated with prevalent fractures before, not after, women with deteriorated trabecular density were excluded. Trabecular density was associated with prevalent fractures before, not after, women with deteriorated cortical porosity were excluded. The coexistence of an increment in cortical porosity and a decrement in trabecular density captured by a high structural fragility score was associated with prevalent fractures.

Cortical porosity was associated with prevalent fractures before excluding women with deteriorated trabecular density (OR 2.30; 95% CI, 1.30 to 4.05; *p* = 0.004), not after (OR 0.96; 95% CI, 0.50 to 1.86; *p* = 0.91). Trabecular density was associated with prevalent fractures before excluding women with deteriorated cortical porosity (OR 3.35; 95% CI, 1.85 to −6.07; *p* < 0.0001), not after (OR 1.60; 95% CI, 0.78 to 3.28; *p* = 0.20). By contrast, the coexistence of an increment in cortical porosity and a decrement in trabecular density captured by a high SFS women was associated with prevalent fractures (OR 4.52; 95% CI, 2.17 to 9.45; *p* < 0.0001) (Fig. 4).

### SFS is associated with fractures independent of BMD and outperforms BMD

As shown in detail in Table 2, BMD had a sensitivity of 10.1% and a specificity of 98.1% giving an OR of 5.79 (*p* = 0.026) before accounting for the contribution of the SFS. However, after accounting for the contribution of the SFS, BMD was no longer associated with fracture, having a sensitivity of 4.04%, a specificity of 99.0%, and an OR of 4.38 (*p* = 0.19). By contrast, the SFS has a sensitivity 35.4%, a specificity of 89.5%, and an OR of 4.67 (*p* < 0.0001) before accounting for the contribution of BMD, and a sensitivity of 29.3%, specificity of 90.5%, and an OR of 3.94% (*p* < 0.0001) after accounting for the contribution of BMD. The results were similar examining women with osteopenia or normal BMD (not shown).

**Table 2:** Sensitivity, Specificity, and Odds Ratios for the Associations Between BMD, the Structural Fragility Score (SFS), and Fracture Prevalence Before and After Accounting for the Contribution of the Other Predictor

Sensitivity (%) (95% CI)		Specificity (%) (95% CI)		Odd ratios (95% CI; p value)	
Before	After	Before	After	Before	After
BMD before and after accounting for the contribution of the SFS					
10.10 (4.95–17.80)	4.04 (1.11–10.00)	98.10 (93.30–99.80)	99.00 (94.80–100)	5.79 (1.24–27.10; *p* = 0.026)	4.38 (0.48–39.90; *p* = 0.19)
The SFS before and after accounting for the contribution of BMD					
35.40 (26.00–45.60)	29.30 (20.60–39.30)	89.50 (82.00–94.70)	90.50 (83.20–95.30)	4.67 (2.20–9.88; *p* < 0.0001)	3.94 (1.80–8.61; *p* = 0.001)

## Discussion

All traits, cortical porosity, trabecular density, BMD, and the SFS were associated with prevalent fractures. However, the association between cortical porosity or trabecular density and fracture was contingent upon the copresence of deficits in the other trait as captured by the SFS, which outperformed BMD.

Early work investigating the microstructural basis of bone fragility focused on trabecular bone because these thin intersecting plates of mineralized bone matrix have a large surface area/matrix volume ratio that facilitates more rapid remodeling and bone loss than cortical bone, which is configured with a smaller surface area/matrix volume and so is remodeled and lost more slowly.^22^ Subsequent research focused attention on cortical porosity as a cause of bone fragility because ∼80% of the skeleton is cortical and ∼80% of all bone loss is cortical. Most of this bone loss is the result of intracortical remodeling, which leaves cortical porosity as a “footprint” of the bone loss.^13^

Measurements of absolute values of cortical porosity and trabecular density do identify women with prevalent or incident fractures,^4^, ^5^, ^6^ but do so inconsistently^9^, ^10^, ^11^, ^12^ for reasons that may partly relate to challenges in image acquisition and analysis.^13^, ^14^ We examined whether a measure of deterioration in both cortical and trabecular bone produces a more sensitive and specific assessment of fracture risk because both cortical and trabecular compartments of bone contribute to bone strength,^7^ and unbalanced remodeling, appearing in midlife upon intracortical, endocortical, and trabecular surfaces, causes concurrent cortical and trabecular bone loss, producing microstructural deterioration and fragility of both compartments.^13^, ^22^, ^23^, ^24^, ^25^, ^26^

We developed an algorithm that quantifies coexisting cortical and trabecular deterioration—the deviation from the nadir of cortical porosity and the deviation from the peak trabecular density achieved in young adulthood and subsequently deteriorated by bone loss. We proposed that expressing the deterioration of these traits captures the microstructural basis of bone fragility and minimizes the contribution of peak microstructure.

We also wanted to ensure that the porosity measured was the result of bone loss, not the void volume formed by intracortical canals assembled during growth. Over 80% of the measured porosity is void volume formed by these canals seen as pores in cross‐section.^17^, ^18^, ^19^ The porosity of interest is the age‐related increment above that achieved during growth. The void formed by canals assembled during growth are a surrogate of bone strength because they are part of the hierarchical structure of the osteon, the bone structural unit. The canal, the surrounding concentric lamellae of mineralized collagen fibers, and the cement line surrounding the osteon and separating it from interosteonal (interstitial) bone form edges or discontinuities, deflecting or obstructing the propagation of microcracks through bone.^20^, ^21^

As another potential source of error, we needed to ensure that there was coexisting cortical and trabecular deterioration because coexisting deficits are more likely to be the result of bone loss than measurement error as discussed in the Introduction and elsewhere.^23^ The data confirm that isolated deficits, high porosity with normal/high trabecular density, or low trabecular density with normal/low porosity were not associated with prevalent fractures.

This study has several limitations. Coexisting deficits with an increment in porosity and decrement in trabecular density are likely to be the result of bone loss, but we cannot entirely exclude a contribution of growth, even though we attempted to minimize any contribution of growth to the deficits observed. Cortical porosity and trabecular density were used to quantify structural deterioration. However, other traits, such as cortical area, thickness, trabecular number, thickness, and separation, as well as matrix mineral density, may each contribute to bone fragility. Further work is needed to explore the role of other traits singly and in combination. Measurements of the unfractured arm were done at a median of 7 months (IQR 3 to 13) postfracture. Changes in microstructure may have followed rather than preceded a fracture, but are likely to be modest and if found to be higher because of greater post‐fracture usage than controls, the observed deficits in cases would be a conservative error.

In summary, unbalanced remodeling at points upon the intracortical surface lining the many Haversian and Volkmann canals produces enlarged, coalesced irregularly shaped cavities, which form stress “concentrators” increasing the focal strain and promoting microcrack propagation.^27^ Unbalanced remodeling upon trabecular surfaces perforate and reduce trabecular density.^25^, ^26^, ^27^, ^28^ The SFS captured deterioration in cortical and trabecular bone and identified women with a prevalent fracture independent of BMD and outperformed current methods such as absolute values of cortical porosity, trabecular density, or BMD alone. The measurement of SFS compliments the measurement of BMD by capturing deterioration in cortical and trabecular microstructure and so identifies women with osteopenia or so‐called normal BMD at risk for fracture who would otherwise be regarded as being at low risk based on their BMD alone. Preliminary studies published in abstract form suggest the assessment of microstructure is likely to identify women with osteopenia at risk for fracture.^29^ Further studies are needed to demonstrate the SFS serves as a surrogate of bone fragility able to identify women at risk, so that treatment can be targeted to those at imminent risk within 1 to 2 years before the first or a subsequent fracture.

## Disclosures

RZ has received grant or research support from Amgen, Asahi, Genzyme; he is a shareholder and a director of the board of Straxcorp. ES has received research support and lecture fees from Amgen, Allergan, Eli Lilly; he is a consultant, director of the board, and shareholder in Straxcorp. YP is an employee of Straxcorp. AG is a shareholder in Straxcorp.
